# Entropic Interactions between Two Knots on a Semiflexible Polymer

**DOI:** 10.3390/polym9020055

**Published:** 2017-02-09

**Authors:** David Richard, Stefanie Stalter, Jonathan Tammo Siebert, Florian Rieger, Benjamin Trefz, Peter Virnau

**Affiliations:** 1 Department of Physics, Johannes Gutenberg University Mainz, Staudinger Weg 9, 55128 Mainz, Germany; davricha@uni-mainz.de (D.R.); ststalte@uni-mainz.de (S.S.); jsiebert@uni-mainz.de (J.T.S.); riegerf@uni-mainz.de (F.R.); benjamin.trefz@uni-mainz.de (B.T.); 2 Graduate School Materials Science in Mainz, 55128 Mainz, Germany

**Keywords:** knots, DNA, free energy barriers

## Abstract

Two knots on a string can either be separated or intertwined, and may even pass through each other. At the microscopic scale, such transitions may occur spontaneously, driven by thermal fluctuations, and can be associated with a topological free energy barrier. In this manuscript, we study the respective location of a trefoil ($3_{1}$) and a figure-eight ($4_{1}$) knot on a semiflexible polymer, which is parameterized to model dsDNA in physiological conditions. Two cases are considered: first, end monomers are grafted to two confining walls of varying distance. Free energy profiles and transition barriers are then compared to a subset of free chains, which contain exactly one $3_{1}$ and one $4_{1}$ knot. For the latter, we observe a small preference to form an intertwined state, which can be associated with an effective entropic attraction. However, the respective free energy barrier is so small that we expect transition events to occur spontaneously and frequently in polymers and DNA, which are highly knotted for sufficient strain lengths.

## 1. Introduction

Knots are part of our history. They were not only used for practical purposes in tool making, agriculture, fishing, and bookkeeping, but also played an important cultural role in literature, ornamentics, jewelry, and ancient rituals [1]. Despite their unquestionable usefulness, the mathematical description of knots (e.g., as opposed to algebra or geometry) is a rather modern phenomenon, and was arguably kickstarted by Lord Kelvin’s hypothesis that atoms are made up of knots in the ether about 150 years ago [2,3]. Interest in knots among natural scientists was revitalized in the 1960s [4,5], when knots were conjectured to appear in polymers and DNA of sufficient length. The subsequent discovery and creation of molecular knots in DNA [6,7,8,9,10,11,12,13], polymers [14,15] and proteins [16,17,18,19,20,21,22,23,24] led to a renaissance of this interdisciplinary field in which computer simulations of prime [23,25,26,27,28,29,30,31,32,33,34,35,36] and composite knots [37,38,39,40,41,42,43,44] played a pivotal role.

**Figure 1:**
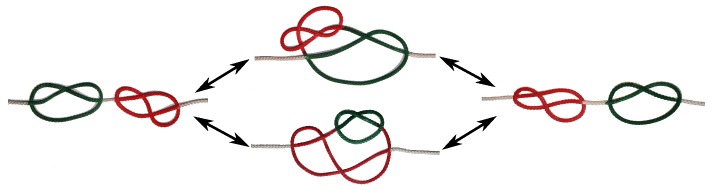
Swapping mechanism. Sketch of a transition event: one of the two knots is embedded in the other knot (**middle**) and diffuses along its contour until the two knots have switched positions (**right**) or returned to their original positions (**left**) along the strand.

The first simulations of random walks in the 1970s [25,26] attempted to map knots onto DNA and determine knotting probabilities of long double-stranded DNA (dsDNA) strands (which turned out to be surprisingly accurate). Modern coarse-grained models of DNA in the context of knotting became available with the first experimentally determined knotting probabilities for DNA (of up to 10,000 base pairs) based on gel-electrophoresis [9,10]. These models map DNA either onto bead-spring(/-stick) [35,42] or cylinder-type models [10], which incorporate stiffness and self-avoidance so that experimental knotting probabilities are matched. Screened Coulomb interactions—which depend on salt conditions—are typically subsumed in the form of an effective diameter, which again influences the stiffness of the chain. Knotting in DNA has recently sparked interest in the context of DNA nanopore sequencing [13,35,37,45], in which single- or double-stranded DNA is pulled through an artificial or biological pore. In such a setup, DNA is typically sequenced by measuring an ionic current through the pore, which is determined by the base pairs located at the bottleneck. Even though knots may be floppy enough to not block the entrance of the pore completely [35,37,45], they may nevertheless influence the ionic current. In fact, the translocation of prime and composite knots highly depends on the particular knot type and the order in which they appear, and not necessarily on their apparent complexity [37]. In recent work, we have unravelled a mechanism which in principle allows two knots on a polymer strand to swap positions by “tunneling” through each other. Even though this appears counterintuitive at first as no bond crossing is allowed, the mechanism can be realized with a macroscopic string (Figure 1). Essentially, one of the knots encompasses the other, while the latter diffuses along the contour of the encompassing knot. Two outcomes are possible: the knot can either leave on the side it has entered, or traverse to the opposite side. However, such a transition is not without cost, and the knot typically has to overcome a free energy barrier, which arises due to topological constraints. Simulations of rather small chains (N=250) attached to two confining walls and containing a trefoil and a figure-eight knot indicate a barrier height of only a few $k_{B}T$ [42], which in principle would allow for an experimental observation of the mechanism for DNA confined between two atomic force microscopy (AFM) tips. In recent publications, Najafi et al. [43,44] extended this work to other combinations of knots (including $3_{1}\#3_{1}$ and $5_{1}\#3_{1}$), and demonstrated that the free energy profile also depends on the relative chirality of the two knots. In Reference [43], the authors focus on the dependence of stiffness on the transition (which was also analyzed in [42]) and analyze free energy contributions due to entropy and bending energy in detail. The latter was shown to become the dominant component in the intertwined state and the transition process. Both studies employ rather small chains (N=300 beads), and the wall distance is fixed to L=205σ, which corresponds to a rather stretched setup. This study is thus complementary, as we investigate the influence of the wall distance on the free energy profile. In addition, we study rather large chains of up to *N* = 1000 beads with and *N* = 5000 beads without confining walls (corresponding to 15,000 and 75,000 base pairs, respectively).

## 2. Model and Methods

### 2.1. Potentials and Mapping onto DNA

We employ a coarse-grained bead spring-polymer model [42]. The chain is modeled by repulsive beads which interact via a Weeks–Chandler–Andersen (WCA) potential:

(1) $$U_{\mathrm{WCA}}\left(r_{ij}\right)=\left\{\begin{array}{cc} 4\epsilon \left({\left(\frac{\sigma }{r_{ij}}\right)}^{12}-{\left(\frac{\sigma }{r_{ij}}\right)}^{6}+\frac{1}{4}\right) & r_{ij}\leq 2^{1/6}\sigma \\ 0 & \mathrm{otherwise}. \end{array}\right.$$

The characteristic length scale of the system is the chain’s diameter *σ*, which is used as a unit of length. The energy scale *ϵ* is set to $1k_{B}T$, which serves as a unit of energy. From here on, both will be omitted for brevity.

In addition to the hard core repulsion, neighboring monomers along the chain are connected by a finitely extensible nonlinear elastic (FENE) bond:

(2) $$U_{\mathrm{FENE}}\left(r_{ij}\right)=\left\{\begin{array}{cc} -\frac{kR_{0}^{2}}{2}\ln\left(1-{\left(\frac{r_{ij}}{R_{0}}\right)}^{2}\right) & r_{ij}\leq R_{0}\;\mathrm{and}\;\left|i-j\right|=1\\ \infty & r_{ij}>R_{0}\;\mathrm{and}\;\left|i-j\right|=1\\ 0 & \mathrm{otherwise}, \end{array}\right.$$

where the parameters for the strength of the bond potential $k=30\epsilon /\sigma^{2}$ and the maximum bond length $R_{0}=1.5\sigma$ are chosen to prevent bond crossings [46], and to thus ensure that the knot types are preserved in Molecular dynamics simulations. In the presence of walls, particles interact with them via a WCA-potential as well:

(3) $$U_{\mathrm{wall}}\left(d_{i}\right)=\left\{\begin{array}{cc} 4\epsilon \left({\left(\frac{\sigma }{d_{i}}\right)}^{12}-{\left(\frac{\sigma }{d_{i}}\right)}^{6}+\frac{1}{4}\right) & d_{i}\leq 2^{1/6}\sigma \\ 0 & \mathrm{otherwise}, \end{array}\right.$$

where $d_{i}$ is the geometrical distance of particle *i* of the respective wall.

Chain stiffness is introduced as a harmonic potential for angles $\theta_{i}$ between adjacent bonds $r_{(i-1)i}$ and $r_{i(i+1)}$ that is centered around *π*:

(4) $$U_{\mathrm{angle}}\left(\theta_{i}\right)=\frac{\kappa }{2}{\left(\theta_{i}-\pi \right)}^{2}.$$

The stiffness of the chain is thus controlled by the parameter *κ*.

To map our model onto DNA, we first match the length scale given by the chain diameter. In our simulations, this is given by *σ*, whereas the diameter of DNA was given as 5 nm for physiological salt concentrations (0.15 M NaCl) in References [10,42]. Hence, one coarse-grained bead roughly corresponds to 15 base pairs (1bp≃0.33nm). Second, we have to tune the energy scale set by *κ* to reproduce the chain stiffness of DNA. This is done by matching the persistence length $l_{p}$ of our model with that of DNA (50 nm) [42]. For an ideal discrete worm-like chain model,

(5) $$\kappa \approx \frac{l_{p}k_{B}T}{\sigma }.$$

The validity of this in the context of our model which allows for variable bond lengths and angles and includes excluded volume interactions has been verified by computation of the persistence length for various values of *κ* from the decay of the bond angle autocorrelation function of a free chain. Therefore, a value of $\kappa \approx \frac{50nm}{5nm}=10$ will allow us to reproduce the chain stiffness of DNA. In previous work [42], we have verified that this parameter set reproduces the experimentally determined knotting probability for DNA of up to 10,000 base pairs.

### 2.2. Constrained vs. Free Chains

In this work, we examine two distinct cases. First, we study chains that are constrained between two walls. Here, the chain’s ends are grafted onto the walls, and the chain is constrained to the volume between them. As there can be no bond crossings and the ends are constrained, such chains will not change their knottedness or the type of the contained knots. All constrained chains were simulated using a Langevin thermostat in molecular dynamics simulations with the CPU version of HOOMD-blue [47,48] and a time step of Δt=0.01. All starting configurations contain the $3_{1}$ and $4_{1}$ knots we want to study. While the walls allow for simple analysis of the chain, the constraints posed by the walls have a serious impact on the dynamics of chains and their knots. This is studied by comparing different wall distances.

Second, we have examined free chains of lengths N= 1000–5000 without any surrounding walls. These were studied using Monte Carlo (MC) simulations. The chains are manipulated, choosing randomly between local MC moves, the pivot algorithm, and generalized MOS moves [49] to generate uncorrelated configurations. These are then filtered using the Alexander polynomial, which will be described in Section 2.3. Since the Alexander polynomial cannot distinguish between the composite knot and more complicated knots (e.g., the $8_{21}$ knot), the configurations were filtered again by use of the HOMFLY polynomial [50,51] before they are finally analyzed as explained in Section 2.3.

### 2.3. Knot Analysis

Mathematically, knots are only well-defined on closed curves, as any open chain could be closed in a way to contain either no knot or an arbitrarily complex one. Therefore, all chains have to be closed before the contained knots can be analyzed. For the chains grafted onto a wall, this can be done unambiguously by connecting the ends of the chain in a semicircle. The semicircle has to be large enough to ensure that the closing line does not intersect with the knotted chain between the walls. By doing so, we ensure that the same knots can be detected throughout the full simulation. Unfortunately, this does not apply to our simulations of free chains. There, we use the algorithm that most closely resembles the non-mathematical understanding of a knot. The ends of the chain are extrapolated outwards by a straight line in the opposite direction of the center of mass of the chain. At a large distance, these new endpoints are then connected [19], resembling the intuitive understanding of a curve being knotted, if pulling both ends outward does not result in a straight line.

The now mathematically well-defined knots on closed curves can be analyzed by computing the Alexander polynomial. Since we only want to distinguish between simple knot types, we can use the somewhat simpler approach described in References [30,52]—namely, computing the product of the Alexander polynomials at points −1.1 and −1/1.1—and determine $\Delta_{p}\left(-1.1\right)=\left|\Delta (-1.1)\times \Delta (-1/1.1)\right|$, which allows us to distinguish all knot types of up to 10 crossings just as well as the full polynomial.

**Figure 2:**
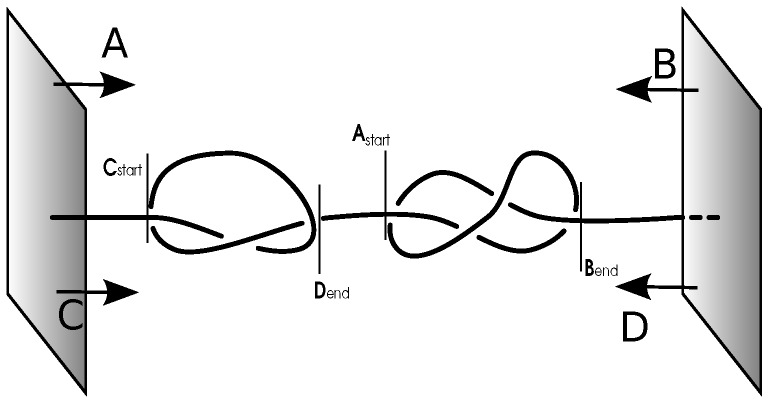
Knots detection. Schematic representation of the knot size analysis. We start by removing beads from the left-hand side (A). As soon as the Alexander polynomial indicates that we have neither composite nor $4_{1}$ knot, the beginning of the $4_{1}$ knot is reached ($A_{\mathrm{start}}$). By repeating this from the right-hand side (B), we get the end of the $4_{1}$ knot ($B_{\mathrm{end}}$). Analogously, for the $3_{1}$ knot, we start from the left-hand side again (C), but check for the Alexander polynomial to be neither composite nor $3_{1}$ knot to get $C_{\mathrm{start}}$. Starting from the other side (D), $D_{\mathrm{end}}$ is obtained.

To study the relative position of the two knots, we have to determine start and end point of the trefoil and the figure-eight knot separately ($3_{1}$ and $4_{1}$ knots were chosen due to their simplicity and the possibility to distinguish them with the Alexander polynomial). Our definition of endpoints is shown schematically in Figure 2. To determine the start/end point of a knot, we start removing monomers from one site, starting with the second monomer and closing the resulting gap by a straight line. After every reduction, the Alexander polynomial is determined. When the removal of a monomer results in an Alexander polynomial that is neither the one of the knot we are analyzing nor the one of the composite knot, we define the monomer that was removed last to be the boundary of the knot on that side. The other end point is found by repeating this analysis starting from the other end of the chain. The boundaries of the other knot are found equivalently. The size and position of the knots is then determined as the monomeric distance and the arithmetic mean of the end point’s monomer numbers, respectively [42] .

## 3. Results

### 3.1. Two Knots Constrained between Walls

Just like the shorter chains (N=250) studied in [42], we analyze the interplay of a $3_{1}$ and a $4_{1}$ knot on a chain of length N=1000 (corresponding to 15,000 base pairs in physiological conditions) constrained between two walls at multiple wall distances (ranging from l=100σ–900σ). As described in Section 2.3, we study the relative position of the knots, as well as their respective sizes. Because of the noise due to large fluctuations caused by the small system sizes and the uncertainties in the knot analysis, all signals were smoothened using a moving window average of length 100. These smoothened time evolutions are then time averaged. Averaging those distributions for at least 100 independent runs of 3 × 10^9^Δ*t* each—where every 10^5^Δ*t* a configuration was written—yields the associated probability distributions.

**Figure 3:**
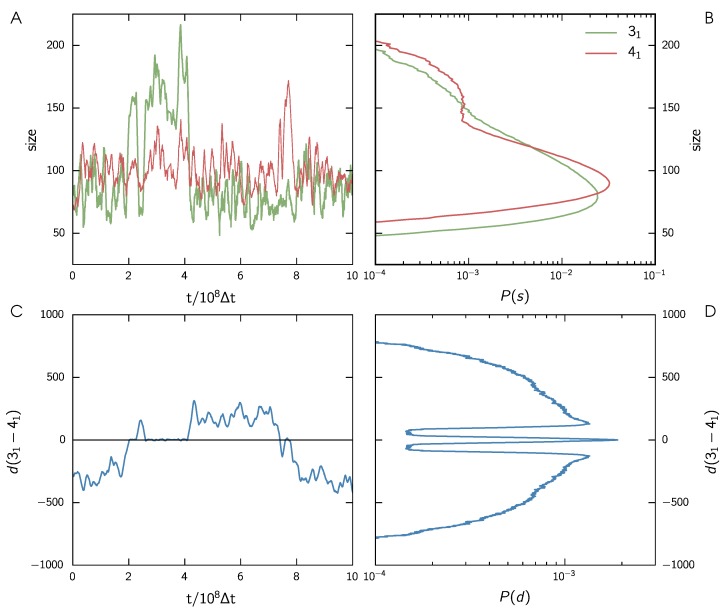
Knot swapping events for wall distance 500*σ*. (**A**) Short excerpt from the time evolution of knot sizes for the trefoil knot $3_{1}$ and the figure-of-eight knot $4_{1}$ in green and red, respectively; (**B**) Probability distribution in log-scale of knot sizes; (**C**) Short excerpt from the time evolution of the distance between the two knots; (**D**) Probability distribution in log-scale of the knot distance.

In Figure 3A, the temporal evolution of knot sizes of the $3_{1}$ and the $4_{1}$ knot for a short time window are shown in green and red, respectively. One can clearly see that the more complex $4_{1}$ knot is usually larger than the simple $3_{1}$ knot. Note also that both knots keep their respective sizes for most of the simulation run. Nonetheless, we can see sharp increases in size for one of the knots in three events along the shown time interval. Around $2\times 10^{8}\Delta t$ and $3\times 10^{8}\Delta t$, the trefoil knot “increases” its size by a factor of 2–3, while the figure-eight knot’s size stays constant. Close to $8\times 10^{8}\Delta t$ the figure-eight knot “grows”, while the size of the trefoil knot remains unchanged. As we measure size in terms of monomers, a size “increase” of the encompassing knot is expected as it also includes the number of monomers of the embedded knot in the intertwined state.

These sharp increases in size are associated with attempted swapping events. This can be seen clearly in Figure 3C. Here, the relative position of the knots is shown for the same time interval as in panel A. Negative values refer to the trefoil being left of the figure-eight knot; positive values arise in the opposite case. Near-zero events occur when the knots are intertwined. When checking the three events we found in the size evolution, we can see that the peaks at $2\times 10^{8}\Delta t$ and $8\times 10^{8}\Delta t$ correspond to successful transition events. Here, the relative position changes sign, which means that the knots changed their positions along the strand. At $3\times 10^{8}\Delta t$, we can also see a long (≈1$\times 10^{8}\Delta t$) but failing attempt at interpenetration. In this case, the relative position is positive before and after the event. Nonetheless, the knots were intertwined during the “growth” of the trefoil knot, indicated by their near-zero relative position.

These observations show that the mechanism proposed in Figure 1 can indeed happen for our chains. The knots contained on the chain spontaneously change positions when one of the knots diffuses along the contour of the other. A failed attempt occurs when the diffusing knot changes directions and leaves the encompassing knot on the side on which it entered.

To quantify the likelihood of these events, we turn to the probability distributions of sizes and relative position that are shown in log-scale in Figure 3B,D, respectively. The size distributions show two distinct states for both knot types, corresponding to a small and a grown state, as described above. In both cases, the more complex figure-of-eight knot is somewhat larger than the trefoil in the corresponding state. The distribution of relative positions shows three sharply separated states. The symmetrical outer states correspond to the separated configurations. There are two peaks, as the knots can change positions. The symmetrical form indicates that there is no preferred side for either knot. In the middle, separated by steep barriers, there is an intertwined state. This state is sharply peaked around zero, and arises if one knot diffused into the other knot. Note that from Figure 3D one can extract the topological free energy as $F\left(d\right)=-k_{B}T\ln\left(P\left(d\right)\right)$, as suggested in [42].

**Figure 4:**
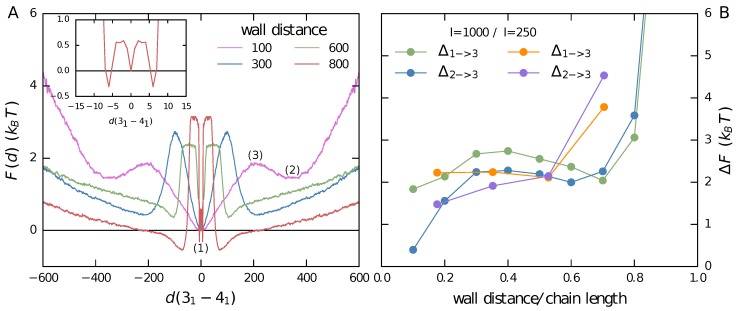
Free energy landscape. (**A**) Free energy landscapes of the knot distance for different end-to-end distances $R_{\mathrm{ee}}$. The inset shows the triple peak structure of the intertwined states at the largest wall distance (800) that was already observed in shorter chains [42]; (**B**) Free energy barriers for different wall distances. The differences in the free energy distribution between the local maximum of the barrier (3) and the local minima corresponding to the intertwined state (1) as well as the separated state (2) are plotted in green and blue for *l* = 1000 and in orange and purple for *l* = 250 [42].

**Figure 5:**
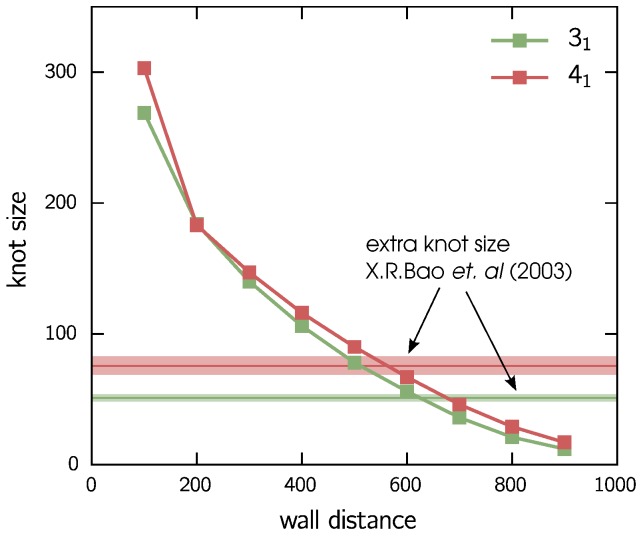
Knot sizes. Evolution of the knot sizes for $3_{1}$ and $4_{1}$ in the separated states. Horizontal lines shows the extra knot length observed experimentally for DNA strand constrained between optical tweezers [53]. Colored area corresponds to the experimental error bars.

As the chains are grafted onto walls with a fixed distance, their end-to-end distances $R_{\mathrm{ee}}$ are fixed. To test the influence of the confining walls, we examined the free energy densities along the order parameter of relative distance for different wall separations and therefore different $R_{\mathrm{ee}}$. For comparison, note that the end-to-end distance of a corresponding free chain is about 140σ. Figure 4A shows the free energy densities for four different wall distances of 100, 300, 600, and 800. To allow for a simple comparison, F(d=0) has been set to zero for all distributions. Several clear trends can be observed. The importance of the separated states (2) increases for increasing end-to-end distances. In the case of $R_{\mathrm{ee}}=100$, the chain is closer to its natural length but slightly compressed. This results in a suppression of the separated states, while the intertwined state (1) shows a broad minimum, which is similar to that of a free chain (see Section 3.2). Thus, compressing the chain between two walls of small distance leads to the knots being intertwined most of the time. Increasing the wall distance to 300 leads to a decreased free energy density for the separated states that becomes intended meaning is retained more probable. Additionally, the minima are shifted to smaller distances. The intertwined state’s minimum becomes more sharply pronounced, and the height of the barrier between the different states increases. At a wall distance of 600, all states’ minima become more pronounced. At the largest simulated wall distance of 800, these trends continue. Here, the free energy density of the separated states actually becomes smaller than that of the intertwined states. At this point, the knots become so small (Figure 5) that they can no longer easily pass through each other. Nevertheless, even in the separated state, they prefer to stay close to each other at a distance which roughly corresponds to their combined sizes. Note also that the middle peak exhibits the triple peak structure that was already found for shorter chains [42]. For better visibility, a zoom of the middle peak at this wall distance is also shown as an inset. The height of the barrier in the free energy density is shown in Figure 4B. Here, we plot the difference in the free energy distribution between the local maximum of the barrier and the local minima corresponding to the intertwined state (Δ_1→3_), as well as the separated state (Δ_1→2_) for long chains (*l* = 1000) and the smaller chains examined in [42]. For both chain lengths, we can see the trend that the height of the barrier separating intertwined (1) and separated states (2) grows with growing wall distance. The steepest increase is observed for small and large wall distances. We also obtain very similar barrier heights for smaller chains (N=250) if the wall distance is normalized by the chain length. Free energy barriers are thus mostly determined by the amount of stretching, and not so much by the chain length itself. The more a chain is stretched, the more the barrier height increases.

The knot sizes in the separated states can also be studied for different wall distances—the results are shown in Figure 5. As one might expect, the average knot size decreases notably for increasing end-to-end distances. This might explain the decreasing distance of the separated knots for larger wall distances. For reference, experimental results for the extra knot length of knots on a DNA strand constrained between optical tweezers [53] are shown as horizontal lines, while the surrounding shaded areas indicate the experimental error bars. For an end-to-end distance of approximately 600, the experimental results match those of our simulated chain constrained between walls. As we observe a barrier height of less than $3k_{B}T$ in this case, it might be possible to observe similar swapping events in an experiment that matches our setup of a $3_{1}$ and a $4_{1}$ knot on the same DNA chain. Due to the simplistic nature of our model, and as all further effects of the wall are neglected, this is to be taken only as a qualitative hint.

### 3.2. Two Knots on a Free Chain

To avoid the influence of confinement, a similar analysis of the interplay of a $3_{1}$ and a $4_{1}$ knot is done for free chains. As these chains are studied by Monte Carlo simulations, which gathers an ensemble average by importance sampling rather than averaging over a time series, all configurations are independent, and time evolutions of the knot positions and sizes are not known. Only ensemble averages of these quantities are accessible. The algorithm to determine the knots’ end points is adjusted slightly. As removal of beads will change the center of mass and thus the closure of the chain (which could influence the knot types), we use the same closing arc during all of the analysis by keeping the end points of the arc fixed. Furthermore, the fluctuations are larger in the case of free chains. Therefore, we change the end point criterium. All chains with an Alexander polynomial that differs from the values of $3_{1}$, $4_{1}$, and composite knot are considered to still carry the composite knot. This minimizes closure effects, where loops on the sides of the chain could be considered as a knot at some intermediate step of the end point analysis.

**Figure 6:**
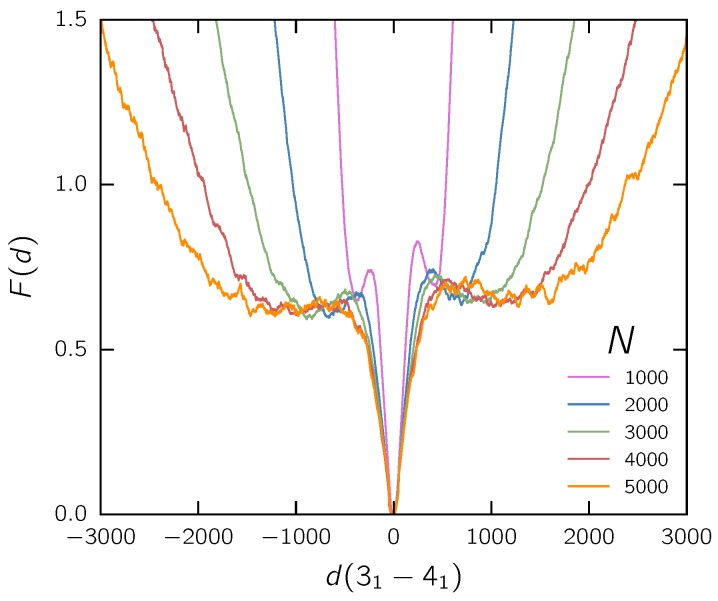
Size effect of free chains. Free energy landscape of the knot distance for different chain lengths composed of *N* beads.

Our results for the free energy density of the relative knot distance for free chains of different lengths are shown in Figure 6. Qualitatively, we get similar results, but the free energy profile is somewhat simpler: the free energy landscape has a global minimum for the intertwined state (at d=0). Analogously to the analysis in Section 3.1, F(d=0) has been shifted to zero for all distributions. As indicated by the global minimum at d=0, the intertwined state is preferred for all chain lengths. The free energy barrier between the local minima corresponding to the separated states diminishes in height for larger chain lengths, while the minima smear out. We also conclude that the rich free energy landscape observed in the confined case mostly arises from the overstretching of the chain. Note that the free energy for knots to separate is lower than $1k_{B}T$ for all chain lengths, and does not change notably for the longer chains that we simulated. Nonetheless, this might indicate that knots attract each other on free chains in the infinitely long chain limit (which is not expected to occur for random walks [40]). In any case, the barrier height is small enough that swapping events should occur almost spontaneously.

## 4. Conclusions

In this work we provide a detailed investigation of a mechanism that allows two knots on a (coarse-grained) DNA strand to swap their respective positions along the strand: one of the two knots diffuses along the contour of the other. In a first step, we investigate chains of size N= 1000 (corresponding to 15,000 base pairs in physiological conditions), which contain a trefoil and a figure-of-eight knot confined between two walls of variable distance to which the termini of the chain are attached. In such a setup, the two knots prefer to stay in an intertwined state unless the chain is overstretched and knots become so small that it is difficult for them to pass through each other. Nevertheless, they still like to stay close to each other. In any case, free energy barriers to enter or leave the intertwined state only amount to a few $k_{B}T$, and should be accessible to an experimental setup in which knotted DNA is confined between two AFM tips. In general, free energy barriers tend to increase with increasing wall distance. In a second setup, we investigated free chains (corresponding to up to 75,000 base pairs) which contain exactly one trefoil and one figure-of-eight knot. Without the confinement of the walls and with no artificial stretching, the free energy profile becomes much simpler and exhibits a small preference (in the order of $0.5\;k_{B}T$) towards an intertwined state, which confines the knots to a rather small portion of the chain, thus enabling the rest of the chain to explore more configurational space. The complex landscape observed in the confined case, however, mostly arises from overstretching the chain. For free chains, we only see a small entropic attraction between the contained knots. At the same time, the barrier to pass through each other is so small that we expect transition events to occur spontaneously and frequently in knotted polymers and DNA.
